# Novel Freshwater Cyanophages Provide New Insights into Evolutionary Relationships between Freshwater and Marine Cyanophages

**DOI:** 10.1128/Spectrum.00593-21

**Published:** 2021-09-29

**Authors:** Dong Zhang, Yiliang He, Karina Yew-Hoong Gin

**Affiliations:** a NUS Environmental Research Institute (E2S2-CREATE), National University of Singapore, Singapore, Singapore; b Department of Civil and Environmental Engineering, National University of Singapore, Singapore, Singapore; c School of Environmental Science and Engineering, Shanghai Jiao Tong University, Shanghai, China; University of Mississippi

**Keywords:** cyanophage, *Synechococcus*, phylogenetic analysis

## Abstract

Cyanobacteria and cyanophages are present widely in both freshwater and marine environments. However, freshwater cyanophages remain unknown largely due to the small numbers of cyanophage isolates despite their ecological and environmental significance. In this study, we present the characterization of two novel lytic freshwater cyanophages isolated from a tropical inland lake in Singapore, namely, cyanopodovirus S-SRP01 and cyanomyovirus S-SRM01, infecting two different strains of *Synechococcus* spp. Functional annotation of S-SRP01 and S-SRM01 genomes revealed a high degree of homology with marine cyanophages. Phylogenetic trees of concatenated genes and whole-genome alignment provided further evidence that S-SRP01 is close evolutionarily to marine cyanopodoviruses, while S-SRM01 is evolutionarily close to marine cyanomyoviruses. Few genetic similarities between freshwater and marine cyanophages have been identified in previous studies. The isolation of S-SRP01 and S-SRM01 expand current knowledge on freshwater cyanophages infecting *Synechococcus* spp. Their high degree of gene sharing provides new insights into the evolutionary relationships between freshwater and marine cyanophages. This relatedness is further supported by the discovery of similar phenomenon from other freshwater viral metagenomes.

**IMPORTANCE** This study expands the current knowledge on freshwater cyanophage isolates and cyanophage genetic diversity, indicating that freshwater and marine cyanophages infecting *Synechococcus* spp. may share close genetic similarity and evolutionary relationships.

## INTRODUCTION

Cyanobacteria play important roles in primary production and trophic interactions. They are the dominant autotrophs in most aquatic environments that originated around 3 billion years ago (1, 2). Across both freshwater and marine environments, cyanobacteria belonging to the genus *Synechococcus* are one of the most abundant and prevalent picophytoplankton (3) with significant ecological roles in primary production.

Viruses infecting cyanobacteria are referred to as cyanophages and can play key roles in the dynamics, genetic diversity, and structure of cyanobacterial communities as well as the biogeochemical cycling of nutrients in aquatic systems (4–7). Cyanophages are categorized generally into 3 families, as follows: *Myoviridae* (with contractile tail), *Podoviridae* (with short tail), and *Siphoviridae* (with long noncontractile tail) (8). Many marine cyanophages have been isolated in the past 2 decades, while relatively few freshwater cyanophage isolates are available (9–14). Cyanopodoviruses and cyanomyoviruses are present widely in the marine environment, and they constitute an indispensable viral fraction in pelagic water (15, 16). A number of marine cyanopodoviruses have been sequenced. Their genomes have similar sizes and core genes (10, 17, 18). Contrary to marine cyanopodoviruses, only 5 freshwater cyanopodoviruses have been isolated (18–21), and they are distant evolutionarily from marine cyanophages (4, 10, 17, 22). Little genomic similarity has been found to be shared among freshwater and marine cyanophage isolates with the exception of T4-like phage S-CRM01 (12, 23). Previous studies indicate few environmental transitions of virus across different biomes, as suggested by distinct viral lineages between marine and freshwater environments (24). A previous study isolated cyanophages from coastal rivers in Georgia with marine *Synechococcus* spp. where the cyanophage abundance increased along the salinity gradient (25). Researchers have also demonstrated using laboratory setups that freshwater viruses can propagate in marine environments (26). Furthermore, a virus demonstrating lytic activity against the marine *Synechococcus* sp. strain WH7803 was found to be present widely in a freshwater lake (27).

Here, we report the isolation and genomic analysis of cyanophage S-SRP01 infecting S*ynechococcus* sp. strain SR-R4S1 and cyanophage S-SRM01 infecting S*ynechococcus* sp. strain SR-C6. Both the host and phage were isolated from a tropical eutrophic freshwater body that is adjacent to the sea but separated by a dam in Singapore. The viruses share core genes and synteny with their marine counterparts. The isolation of cyanophages with a marine genotype in freshwater indicates that marine and freshwater cyanophages infectin*g Synechococcus* spp. may draw from a common gene pool.

## RESULTS AND DISCUSSION

### Morphology.

**FIG 1:**
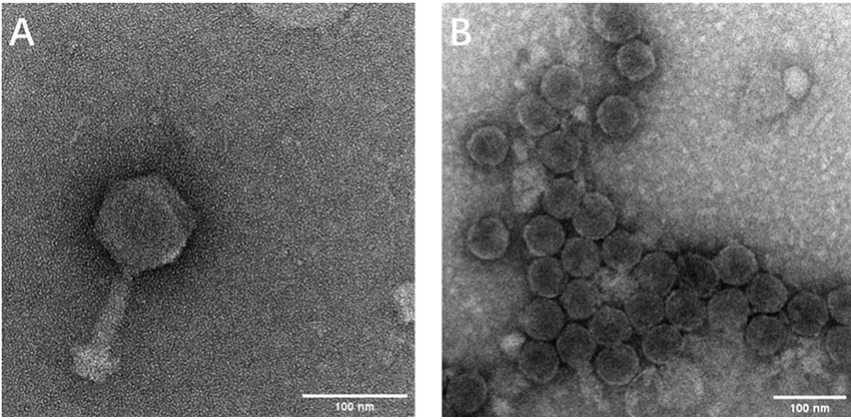
Transmission electron microscope image of S-SRM01 (A) and S-SRP01 (B).

The purified S-SRM01 particle has a polyhedral shape with head diameter of approximately 85 nm (Fig. 1A). A contractile tail sheath of ∼100 nm is attached to the head revealing its morphology characteristic of *Myovirus* (8). Transmission electron microscopy (TEM) of purified S-SRP01 particle revealed its icosahedral shape with a diameter of approximately 50 nm (Fig. 1B). A short tail is visible for virion particles when viewed at the correct angle. S-SRP01 is similar morphologically to previously isolated cyanopodovirus (14).

### Host specificity.

S-SRP01 lysed only *Synechococcus* sp. strain SR-R4S1 and, thus, has a narrow host range (Table 1). This finding agrees with previous studies that showed cyanopodoviruses are highly host specific (14). Similarly, no successful infection was found when other cyanobacteria were challenged against the S-SRM01. To detect for presence of CRISPR sequences in the cyanobacteria used for the host range test, 6 cyanobacteria strains with genome sequences available were submitted to the DFAST server for the identification of CRISPR sequences (28). No CRISPR sequences were found in *Synechococcus* SR-R4S1 and SR-C6, while 2 CRISPR sequences were identified in *Synechococcus* SR-C21. The presence of the CRISPR system in SR-C21 may provide protection against infection by S-SRP01 and S-SRM01 (29). However, further examination of spacer sequences in SR-C21 did not identify any match with S-SRP01 or S-SRM01 nucleotide sequences (E values, <10^−5^). The result suggests there may be other unknown defense mechanisms that protect the tested cyanobacteria host against S-SRP01 and S-SRM01 infection.

**TABLE 1:** List of cyanobacteria used for host range test

Genus or species	Strain	Origin	Susceptibility to S-SRP01	Susceptibility to S-SRM01
*M. aeruginosa*	CS569	Australian National Algae Culture Collection (CRISO) culture collection	**−**	**−**
Anabaena cylindrica	CS172	CRISO culture collection	**−**	**−**
*Cylindrospermopsis*	CS511	CRISO culture collection	**−**	**−**
Anabaena circinalis	CS337	CRISO culture collection	**−**	**−**
*Synechococcus*	SR-C1	Singapore freshwater lake	**−**	**−**
*Synechococcus*	SR-C21	Singapore freshwater lake	**−**	**−**
*Synechococcus*	SR-C6	Singapore freshwater lake	**−**	**+**
*Synechococcus*	SR-R4S1	Singapore freshwater lake	**+**	**−**
*Microcystis*	SR-I31	Singapore freshwater lake	**−**	**−**
	SR-I1	Singapore freshwater lake	**−**	**−**
*Limnothrix*	SR-Fila1	Singapore freshwater lake	**−**	**−**
*Pseudanabaena*	SR-C8	Singapore freshwater lake	**−**	**−**
*Cylindrospermopsis*	CY2.2	Singapore freshwater lake	**−**	**−**
*Pseudanabaena*	M6A	Marina reservoir	**−**	**−**
Nostoc punctiforme	ATCC 29133	American Type Culture Collection	**−**	**−**

### One-step growth curve and adsorption assay.

**FIG 2:**
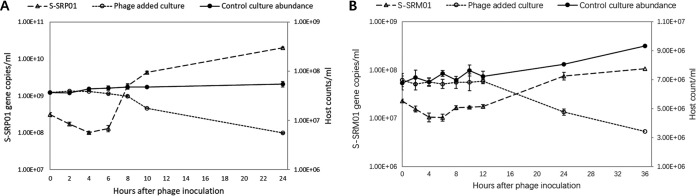
The growth of host *Synechococcus* strain SR-R4S1 (A) and SR-C6 (B) is shown. Host cell abundance was measured by flow cytometry, and free phage abundance was measured by qPCR. Each set up was performed in biological triplicates.

As indicated by the free S-SRP01 abundance profile (Fig. 2A), maximal adsorption of S-SRP01 to its host SR-R4S1 occurred at 4 hours postinfection. However, 1.3 × 10^8^ S-SRP01 has been adsorbed onto the host at 2 hours postinoculation; on average, each SR-R4S1 host could have 3.3 phages attached. The latent period of S-SRP01 was between 6 and 8 hours as free S-SRP01 started to be released from 6 to 8 hours postinoculation. A drastic drop in host abundance was observed from 8 hours postinoculation, and at 24 hours, more than 80% of the host was lysed in the culture with phage added than that of to the control. The burst size was estimated to be ∼600. This is rather a large burst size for cyanophages. However, a similar burst size was also observed with freshwater cyanophage S-LBS1 (30).

Growth curve of S-SRM01 is like S-SRP01 (Fig. 2B). Maximal adsorption occurred at 4 h postinoculation. On average, each SR-C6 host has 24 phage particles attached. The latent period of S-SRM01 is between 6 and 8 hours as free S-SRM01 started to release between 6 and 8 hours postinoculation. At 24 hours postinoculation, over 30% of host cyanobacteria had been lysed, and this number increased to 50% at 36 hours postinoculation. The burst size is estimated to be approximately 30. The much lower burst size is likely due to the huge genomic size of S-SRM01.

### S-SRP01.

**FIG 3:**
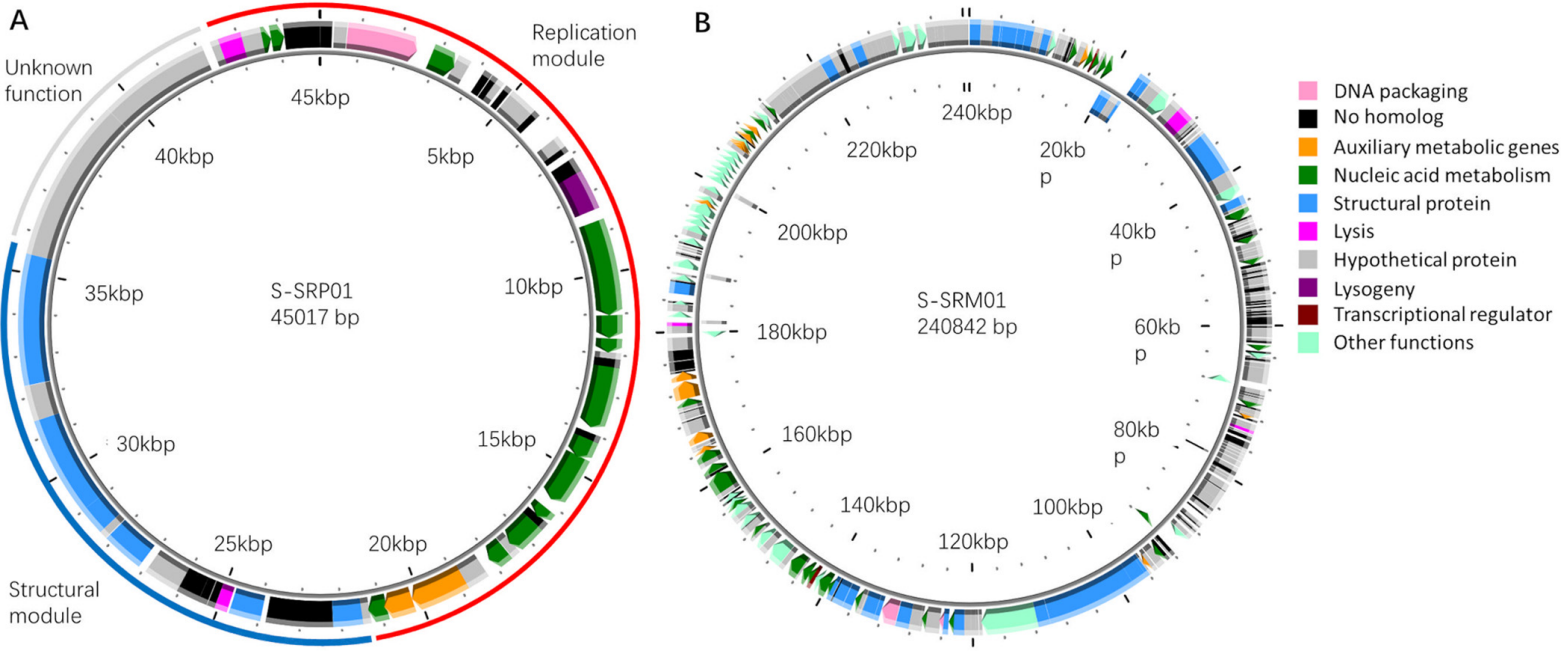
Genomic map of S-SRP01 (A) and S-SRM01 (B). The outer circle corresponds to defined genomic modules, and the inner circle corresponds to predicted coding sequences on the forward strand.

The 45,017-bp genome of S-SRP01 is double stranded with a GC content of 48.9%. This genome length corresponds to the genome size of other cyanopodovirus isolates (10, 14, 19). The high sequencing coverage of ∼40,000 suggests the S-SRP01 genome to be complete. In total, 57 open reading frames (ORFs) were predicted with 32 ORFs which could not be assigned putative function. A total of 21 ORFs with putative function were predicted using Diamond (see Table S4 in the supplemental material). We searched for distant homologs with HHpred and ascribed functions for 4 ORFs that could not be annotated for putative function with Diamond (Table 2). Overall, the 25 ORFs with putative function could be divided into 6 groups, as follows: DNA packaging, auxiliary metabolic genes, nucleic acid metabolism, structural protein, lysis, and lysogeny (Fig. 3A). From the genome organization, we can see that the S-SRP01 genome can be divided into structural module, replication module, and module of unknown function. Interestingly, all ORFs in the S-SRP01 genome are transcribed in one direction with no reverse gene presence. A similar arrangement was observed in P-SSP7 where all genes are transcribed in one direction. P-SSP7 was proposed to have a circular genome based on the sequencing results (17). However, sequencing of digested (with the BamHI and PmeI restriction enzymes) and undigested P-SSP7 DNA revealed a 206-bp terminal repeat, indicating the P-SSP7 genome to be a linear molecule (31). S-SRP01 is likely to have a linear genome, but a further enzyme digestion experiment is needed.

**TABLE 2:** Distant homologs of S-SRP01 and S-SRM01 predicted by HHpred

ORF	Homolog	E value	Function	Phage
25	PF02945.15	2.50E-11	Endonuclease VII (ENDOVII) from Phage T4	S-SRP01
33	3GOX_B	3.00E-21	Restriction endonuclease Hpy99I	S-SRP01
52	5DN5_A	2.30E-14	Glycoside hydrolase	S-SRP01
55	PF13392.6	2.50E-07	Endo-deoxy-ribonucleases	S-SRP01
37	5IV5	5.6E-12	Baseplate wedge protein	S-SRM01
53	PF05268	3.9E-07	gp37-gp38 adhesin tip complex	S-SRM01
66	3LDY	1.2E-10	HNH restriction endonuclease	S-SRM01
302	5UJ0	3.2E-10	T4Pnkp 3′ phosphatase	S-SRM01
303	PF05830	7.1E-11	Fucosyltransferase NodZ	S-SRM01
305	PF10111	2.7E-15	Glycosyltransferase-like family 2	S-SRM01
310	PF13641	2.6E-08	Glycosyltransferase-like family 2	S-SRM01

### S-SRM01.

S-SRM01 is a double-stranded genome of 240,842 bp in length with a GC content of 35.6%. This genome size is comparable but relatively larger than that of other cyanomyoviruses (23, 32, 33). In total, 353 ORFs were predicted with 108 ORFs (see Table S5 in the supplemental material) that could be assigned putative function. A total of 174 ORFs were homologous to hypothetical proteins, while 71 ORFs did not have significant homologs. We searched for distant homologs with HHpred and ascribed functions for 7 ORFs that could not be annotated for putative function with Diamond (Table 2). Overall, the 115 ORFs with putative function could be divided into 7 groups, as follows: DNA packaging, auxiliary metabolic genes, nucleic acid metabolism, structural protein, lysis, transcriptional regulator, and other functions (Fig. 3B).

No tRNA genes were identified in the S-SRP01 genome. Eleven tRNA genes were identified in the S-SRM01 genome and of those most were clustered with at least another tRNA gene (Table 3).

**TABLE 3:** List of tRNA genes identified in S-SRM01

tRNA no.	Nucleotide position		tRNA type	Anticodon
	tRNA begin	Bounds end		
1	33489	33561	Met	CAT
2	33604	33676	Leu	TAA
3	165211	165283	Ala	TGC
4	165288	165368	Leu	TAG
5	184119	184192	Arg	TCT
6	184278	184350	Asn	GTT
7	184559	184631	Thr	TGT
8	184709	184782	Gly	TCC
9	186786	186857	Val	TAC
10	188240	188326	Ser	TGA
11	190503	190578	Pro	TGG

Morphological observation of S-SRP01 proves that it is a cyanopodovirus. This finding is also reflected in the genes shared between S-SRP01 and marine cyanopodovirus isolated from estuaries of Chesapeake Bay. As seen in Table S4, approximately 20% (13) of S-SRP01 ORFs share best-hit with protein sequences from S-CBP3 and S-CBP4. Similarly, significant gene sharing was observed in the S-SRM01 genome with cyanomyoviruses, such as P-SSM2 (19 ORFs with best-hit) and S-PM2 (9 ORFs with best-hit) (Table S5). This result agrees with the observed *Myoviridae* morphology of S-SRM01 under TEM.

### Host-derived auxiliary metabolic genes.

There is a relatively small amount of host-derived auxiliary metabolic genes in the S-SRP01 genome. MazG nucleotide pyrophosphohydrolase is encoded by ORF29. Bacterial MazG has been associated with the regulation of programmed cell death through decreasing cellular (p)ppGPP levels. Cyanophage-carried *mazG* can regulate (p)ppGPP levels to imitate a nutrient deplete condition. This process may direct the host cell toward a state more suitable for macromolecule production, thus facilitating phage replication (34). A recent study examining the role of cyanophage-carried *mazG* showcased that the cyanophage MazG protein hydrolyses dCTP and dGTP preferably (35). Nonetheless, *mazG* could play important roles in the degradation of host DNA to provide monomers for phage DNA replication.

Another host-derived gene in the S-SRP01 genome is *nrdA/nrdB* (ORF31 and ORF32) encoding for putative ribonucleotide reductase. *nrdA/nrdB* carries an important function in the reduction of ribonucleotide diphosphate to produce DNA precursors (36). Cyanophage-carried *nrdA/nrdB* may play a key role in degradation of the host DNA and provision of DNA monomers for phage genome replication. Researchers believe that *nrdA/nrdB* could help enable rapid cyanophage lytic activity (37). This finding corresponds with the short latent period of S-SRP01 of 8 hours.

*nrdA/nrdB* was also found in the S-SRM01 genome. Other than *nrdA/nrdB*, genes commonly found in marine cyanomyoviruses were also present in the S-SRM01 genome (38). Auxiliary metabolic genes related to photosynthesis in S-SRM01 include photosystem II D1 protein (ORF319 and ORF321), highlight-inducible protein *hli* gene (ORF316), ferredoxin *petF* gene (ORF315), and *S*-adenosylmethionine decarboxylase *SpeD* (ORF190) gene. Both ORF319 and ORF321 were predicted to be partial *psbA* genes. Other auxiliary metabolic genes in the S-SRM01 genome include ORF25 that carried the phosphate-inducible gene *PhoH* and ORF135 carrying the carbon metabolic regulator *CP12*.

It is likely that ORF319, ORF320, and ORF321 are involved collectively in the expression and function of the photosystem D1 protein in S-SRM01. It was reported previously that self-splicing group I intron in the *psbA* gene played a key role in upregulating *psbA* expression under high light conditions (39). The lack of a single gene encoding a complete photosystem D1 protein in S-SRM01 may be attributed to the presence of a self-splicing intron that contains an endonuclease since ORF320 is predicted to encode endonuclease and is flanked by a noncoding region of 93 bp upstream and 105 bp downstream (40). A similar arrangement was also found in cyanophage S-CBWM1 where the *psbA* contains a group 1 intron holding a 633-bp ORF which encodes the homing endonuclease (41).

### Phylogenetic analysis.

**FIG 4:**
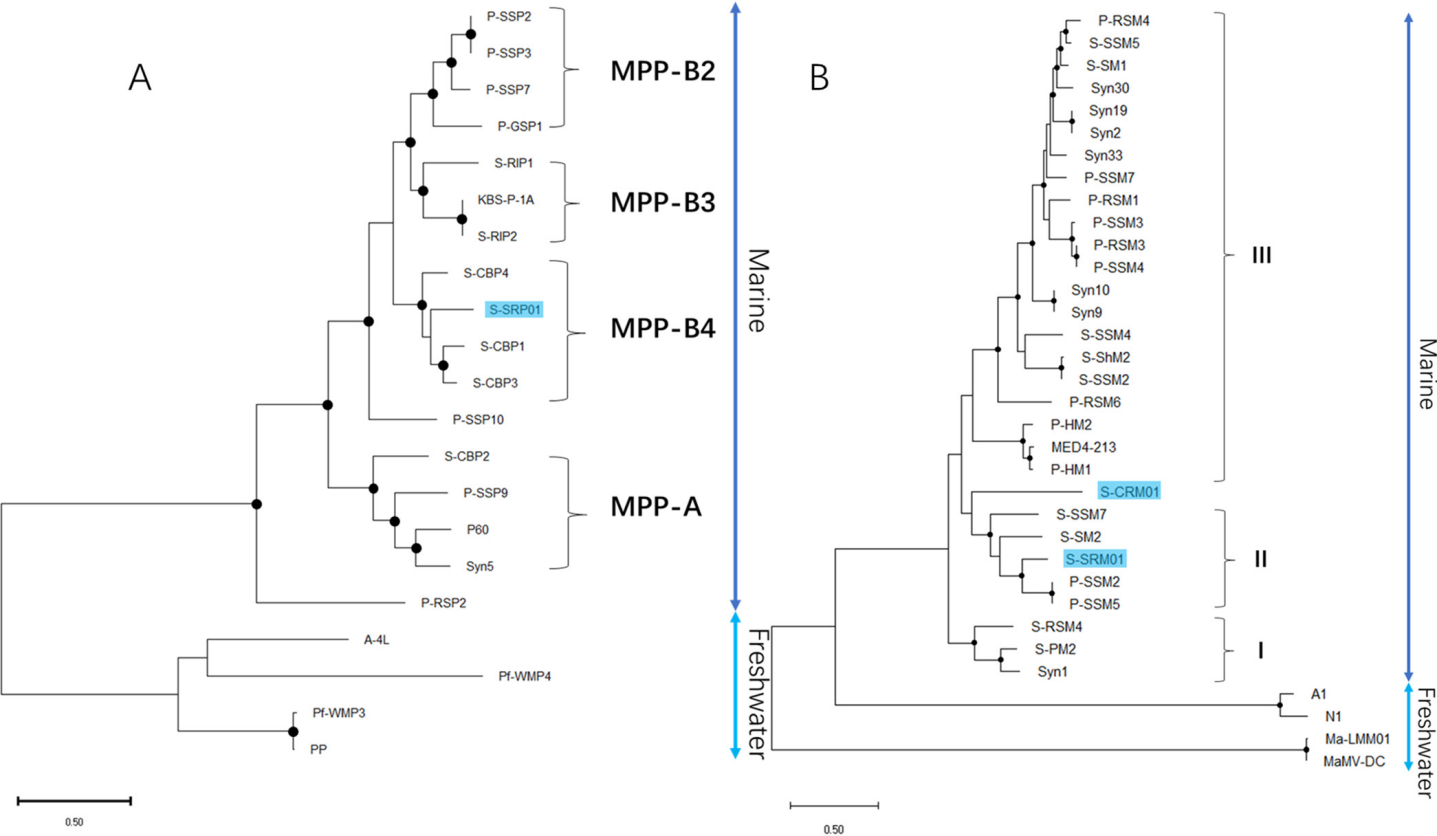
Maximum likelihood tree of inferred amino acid based on concatenated cyanopodovirus (A) and cyanomyovirus (B) genes. Bootstrap values are indicated as black (75% to 100%) dots at the nodes (100 replicates). Cyanophage isolated from freshwater but clustered with marine phages are highlighted in blue.

Terminase large subunit (*Terl*) genes have been used widely for comparing cyanophage phylogenies in various studies (13, 20, 30, 42). DNA polymerase genes and major capsid genes were also used as marker genes in many studies to compare the cyanophage community (43, 44). To have a comprehensive comparison of evolutionary relationships among cultured cyanopodoviruses, a concatenated phylogenetic tree of terminase, DNA polymerase, major capsid protein, and endonuclease was utilized. The concatenated phylogenetic tree of cyanopodovirus (Fig. 4A) revealed the evolutionary relationship of S-SRP01 with other cyanopodoviruses. Generally, freshwater and marine cyanopodoviruses cluster in distinct groups. S-SRP01 is the first freshwater cyanopodovirus clustering with marine cyanopodoviruses despite being isolated from a freshwater lake. It belongs to the MPP-B4 cluster of marine cyanopodoviruses defined previously (45).This result indicates that the genetic distinctness between the freshwater and marine cyanophage community may be overestimated. Freshwater and marine cyanophage may draw from a common gene pool as suggested in previous studies (23).

Phylogenetic analysis of cyanomyoviruses was modified slightly by replacing the major capsid protein with the tail sheath protein, as some freshwater cyanomyovirus, such as MA-LMM01, lack the major capsid gene (32). Corresponding to the significant gene sharing observed in functional annotation, S-SRM01 clustered closely with marine phage P-SSM2 and P-SSM5 and belongs to the previously defined cluster II of marine cyanomyoviruses (46). Cyanophage S-CRM01 was isolated previously from the Klamath River and found to be present in the Klamath River (23). It shared a close phylogenetic relationship to marine cyanomyoviruses, and this relationship is observed in Fig. 4B. The discovery of S-SRM01 provides further evidence that some freshwater and marine cyanomyoviruses may draw from a common gene pool.

To further explore the phylogenetic relationship of these two phages, ViPTree was used to construct a virus proteomic tree comparing S-SRP01, S-SRM01, and 2620 dsDNA phages deposited in the Virus-Host Database (https://www.genome.jp/virushostdb/) (see Fig. S3 in the supplemental material). The result agrees with the phylogenetic tree constructed in this study.

### Comparative genomics.

**FIG 5:**
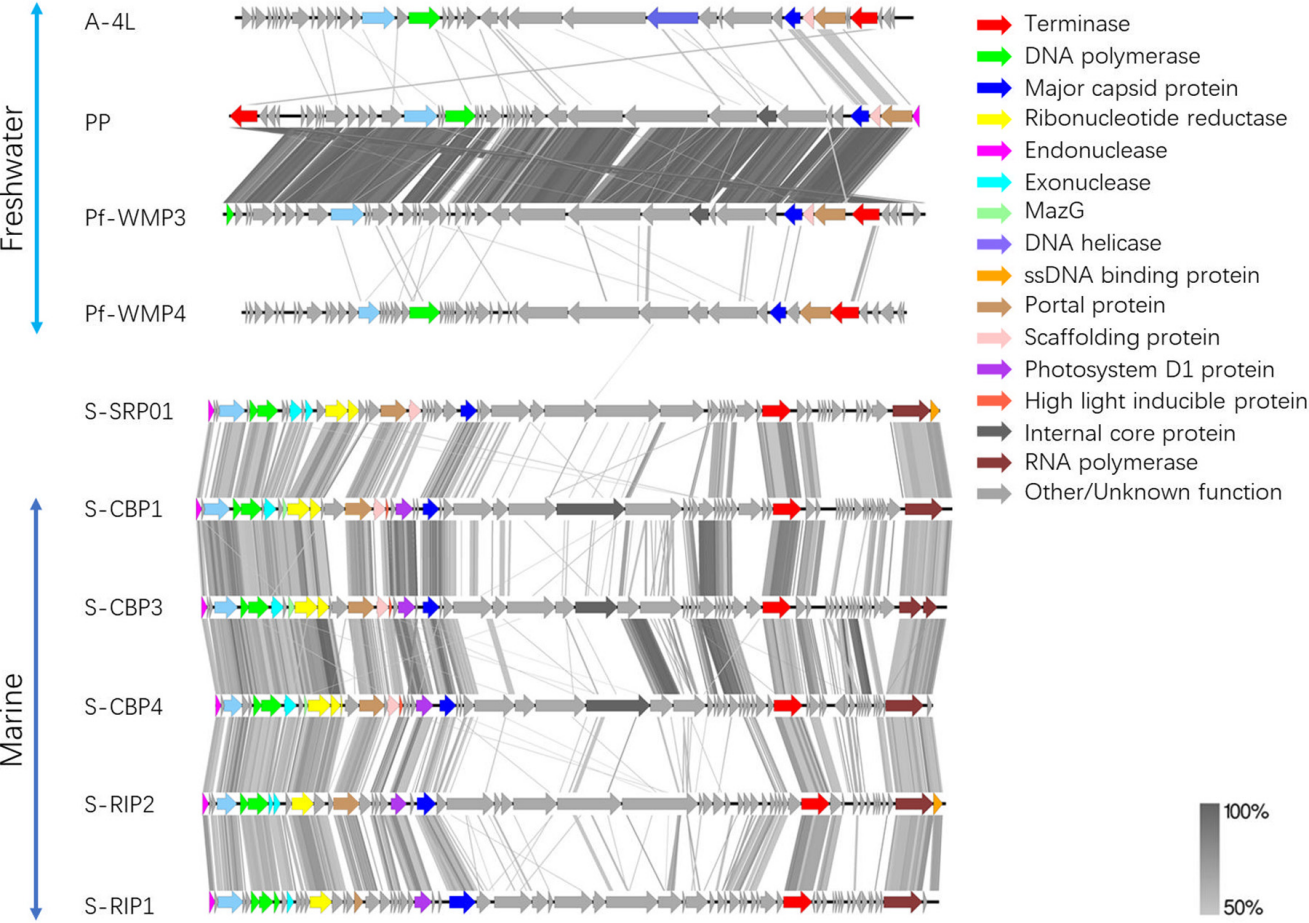
Whole-genome protein sequence alignment of cyanopodoviruses. Colored arrows correspond to predicted genes of a particular function. Gray bars correspond to tBLASTx matches with a minimum percentage identity of 50%.

Genome-wide protein-based comparisons of S-SRP01 and cyanopodoviruses further confirmed that S-SRP01 has a high degree of relatedness to cyanopodoviruses with estuarine origin (Fig. 5). Significant gene sharing and synteny were observed between S-SRP01 and S-CBP1, S-CBP3, S-CBP4, and S-RIP1 as well as S-RIP2. A total of 14 out of 15 marine cyanopodovirus core genes could be identified in the S-SRP01 genome with the exception of the tail fiber protein (10). On the other hand, little gene sharing could be observed between S-SRP01 and freshwater cyanopodoviruses, probably due to the fact that available freshwater cyanopodoviruses target different species of cyanobacteria. S-EIV1 is a freshwater cyanopodovirus infecting *Synechococcus* sp. strain PCCC-A2c; however, it forms its very own evolutionary lineage, lacks many core genes found in cyanopodovirus, and is therefore unsuitable for phylogenetic trees and comparative genomics in this study (20).

**FIG 6:**
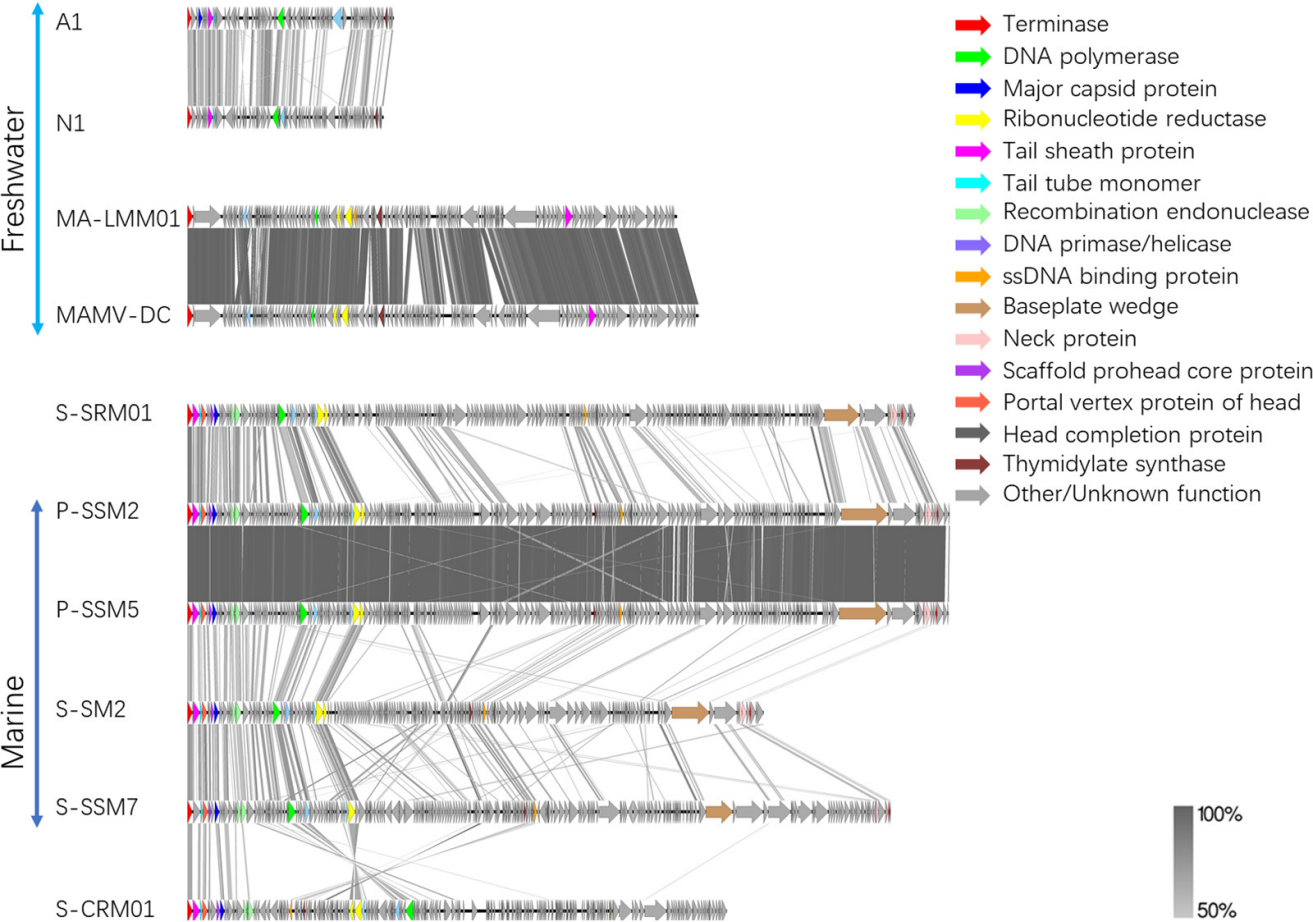
Whole-genome protein sequence alignment of cyanomyoviruses. Colored arrows correspond to marine cyanomyovirus core genes of a particular function. Gray bars correspond to tBLASTx matches with minimum percentage identity of 50%.

A similar trend was also observed in the genome-wide protein-based comparisons of S-SRM01 with other cyanomyoviruses (Fig. 6). Significant gene sharing can be seen between S-SRM01 and P-SSM2. S-SRM01 was shown to be closer phylogenetically to P-SSM2. P-SSM5 and P-SSM2 shared almost identical genes. A higher degree of gene synteny could be observed between S-SRM01 with P-SSM2 than that between S-SM2 and P-SSM5, as indicated by the dense gray lines representing tBLASTx hits (Fig. 6). On the other hand, not a single gene homology meeting the cutoff value of 50% identity could be observed between S-SRM01 with freshwater cyanomyovirus MAMA-DC. Such a dissimilarity in genomic content may be attributed to MAMA-DC infecting different host cyanobacteria from S-SRM01. With more freshwater cyanomyoviruses infecting *Synechococcus* sp. available in the future, a clearer picture could be drawn.

### S-SRP01 and S-SRM01: possible origins.

Genetic contents, phylogenetic analysis, and comparative genomics suggest S-SRM01 and S-SRP01 might be of marine origin; yet, they were isolated from freshwater environments and cultured using freshwater *Synechococcus* sp. grown in MLA medium. The freshwater lake from which they were isolated is separated from the adjacent coastal waters by a tidal gate. The close proximity between the lake and sea, coupled with occasional release of freshwater into the sea when the gate is opened (e.g., after a storm), could have allowed mixing of seawater with freshwater. It is possible that S-SRP01 and S-SRM01 originated from these fresh water and seawater mixing events, suggesting the marine cyanophage adapts to the freshwater environment. Nonetheless, there is a lack of evidence for the marine origin of S-SRP01 and S-SRM01 due to the very limited availability of freshwater cyanophages. Phages such as S-SRP01 and S-SRM01 could also be present naturally in freshwater environments.

Another possibility is that instead of cyanophage adaptation alone, they coadapted to the freshwater environment with their host cyanobacteria. Indeed, phylogenetic trees based on host cyanobacterial 16S sequences showed that *Synechococcus* strain SR-R4S1 and strain SR-C6 displayed some genetic proximity to marine *Synechococcus* spp. (47). However, there is insufficient evidence to show their marine origin, and further study is needed to evaluate this possibility.

### Detection of cyanophage gene sharing in the metagenome.

**FIG 7:**
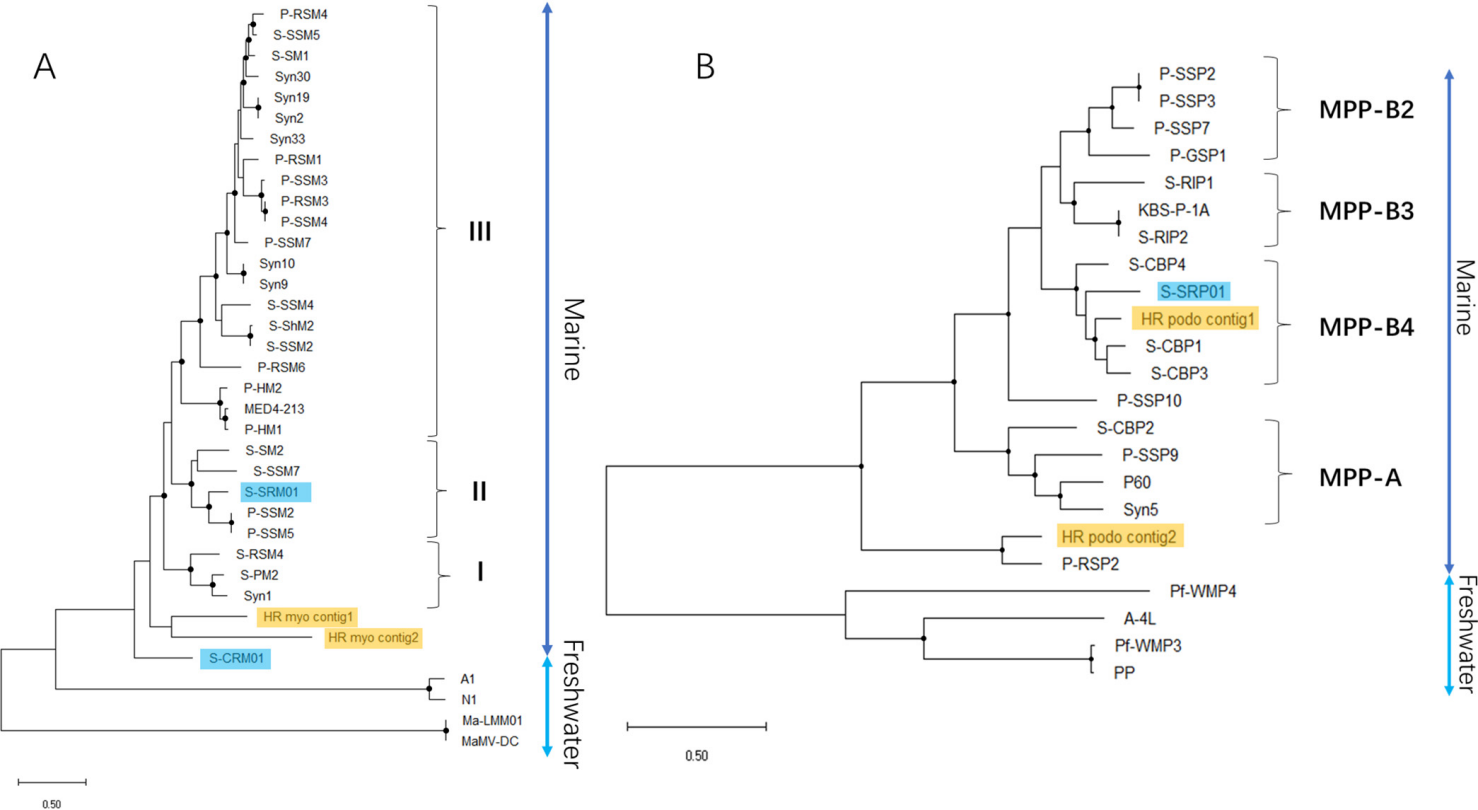
Maximum likelihood tree of inferred amino acid based on concatenated cyanopodovirus gene (A) and cyanomyovirus gene (B) with viral contigs assembled from environmental viral metagenome. Bootstrap values are indicated as black (75% to 100%) dots at the nodes (100 replicates). Cyanophage isolated from freshwater but clustered with marine phages are highlighted in blue, and cyanophage contigs assembled from freshwater viral metagenome are highlighted in yellow.

To find out whether a such degree of gene sharing between marine and freshwater cyanophages is prevalent, we examined the presence of marine cyanophages by genome assembly with some published freshwater viral metagenomes. In total, 4 viral contigs sharing at least 10 homologous genes with marine cyanopodoviruses were identified preliminarily. Two viral contigs were from Han River, South Korea (HR), and the other 2 were from freshwater lakes of Chattahoochee River, United States (CR). The 2 selected contigs from Han River were used for phylogenetic analysis, as described above for cyanopodoviruses (Fig. 7A). Due to a lack of necessary genes, contigs from Chattahoochee River were not used for the construction of the phylogenetic tree. Surprisingly, HR podo contig 1 identified from the Han River metagenome is closer evolutionarily to S-CBP1 than our isolate S-SRP01 and belongs to the MPP-B4 cluster. On the other hand, HR podo contig 2 clusters with marine cyanopodovirus P-RSP2 which forms a distinct clade of marine cyanopodovirus. Although there are limitations in the number of data sets analyzed, these initial results suggest that gene sharing between freshwater and marine cyanophages infecting *Synechococcus* spp. could be more prominent than expected in the environment.

**FIG 8:**
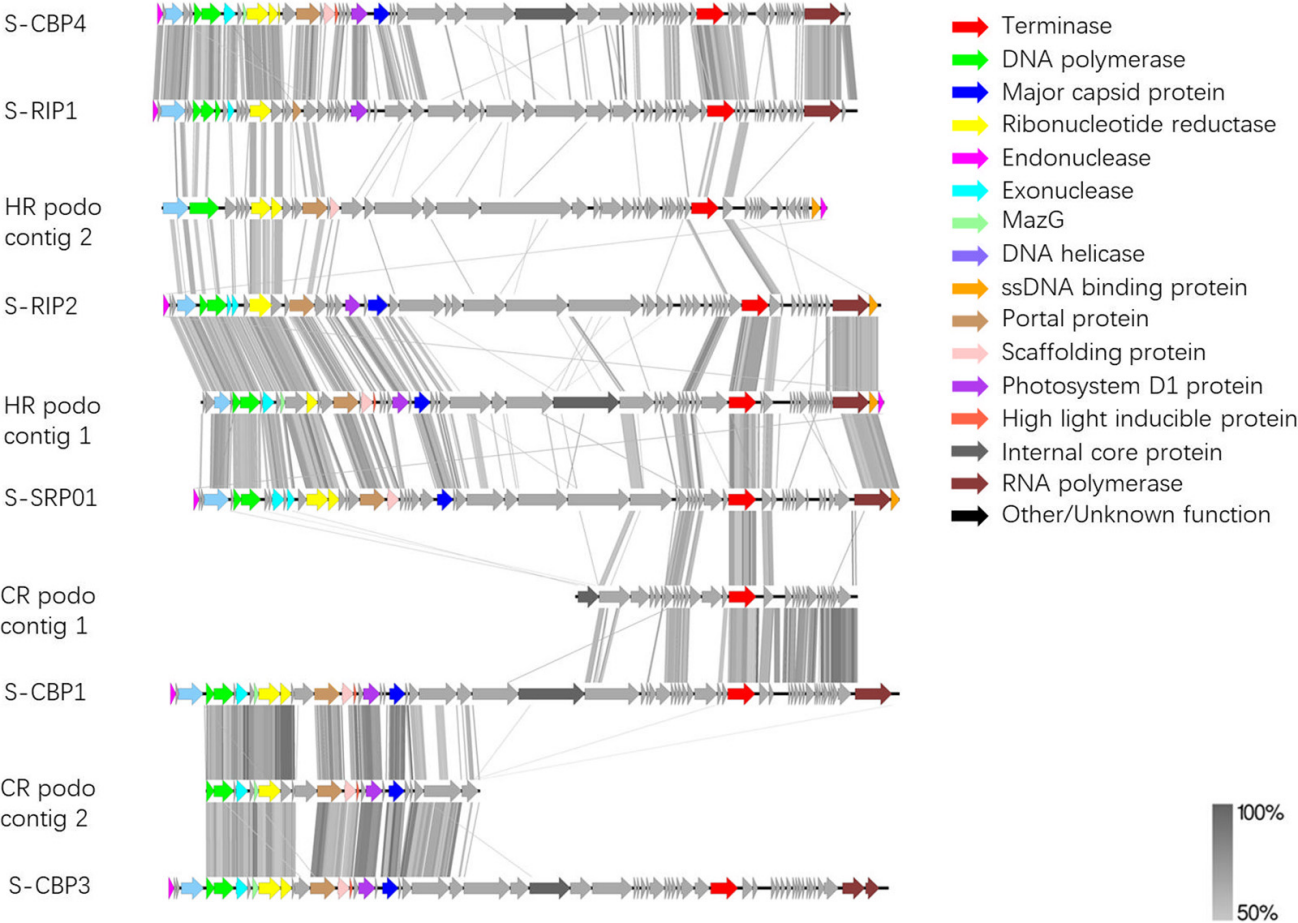
Whole-genome protein sequence alignment of cyanopodoviruses with viral contigs assembled from freshwater viral metagenome. Colored arrows correspond to predicted genes of a particular function. Gray bars correspond to tBLASTx matches with minimum percentage identity of 50%.

The whole-genome protein sequence alignment (Fig. 8) demonstrates that HR podo contig 1 shares significant similarity and gene synteny with marine cyanopodoviruses as well as S-SRP01. In comparison, HR podo contig 2 is more divergent from marine cyanopodoviruses, and yet, approximately 40% (19 ORFs) of HR podo contig 2 genes are homologous to marine cyanopodovirus ORFs. Unlike contigs from HR, CR contigs seem to be partial phage genomes as indicated by the genome size and component. CR podo contig 1 is rich in host-derived genes and lacks genes related to nucleic acid metabolism. On the other hand, CR podo contig 2 is rich in host-derived genes and genes related to nucleic acid metabolism while lacking structural genes and DNA packaging genes. Although the complete cyanopodovirus genome sequence could not be obtained, CR podo contig 1 and CR podo contig 2 share tremendous gene synteny with marine cyanopodoviruses. As indicated by the alignment color, genes of both contigs share high percentage identity with the marine cyanopodovirus (Fig. 8).

**FIG 9:**
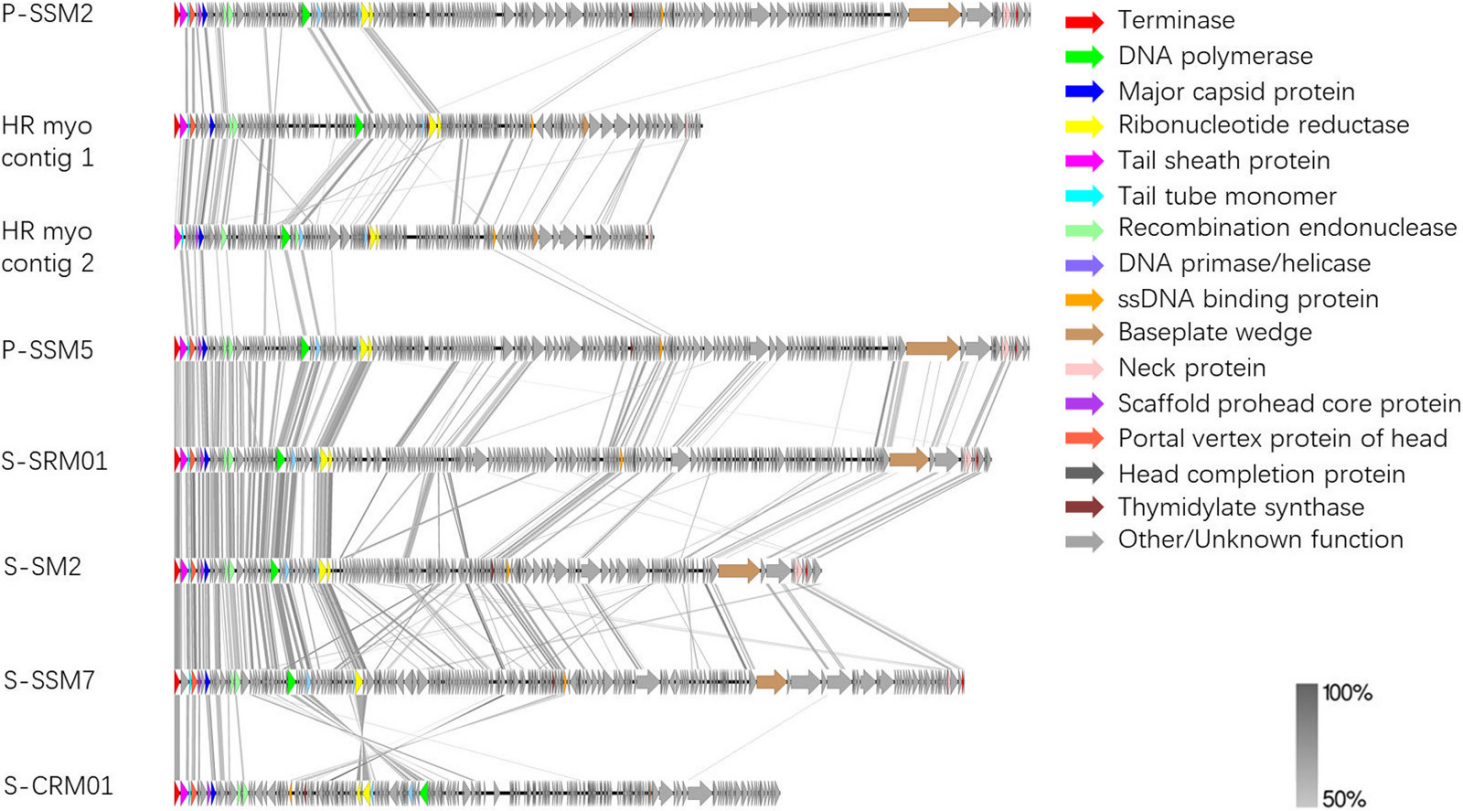
Whole-genome protein sequence alignment of cyanomyoviruses with viral contigs assembled from freshwater viral metagenome. Colored arrows correspond to marine cyanomyovirus core genes of a particular function. Gray bars correspond to tBLASTx matches with minimum percentage identity of 50%.

For cyanomyoviruses, similar findings were also observed in the Han River metagenome where 2 viral contigs identified from Han River were found to share a closer evolutionary relationship with marine cyanomyoviruses than freshwater ones, and they do not belong to the major marine cyanomyovirus clusters defined previously (Fig. 7B). Although the degree of gene sharing between HR cyanomyovirus contigs were less prominent than those observed in cyanopodovirus, they shared a majority of marine cyanomyovirus core genes (Fig. 9).

Discovery of cyanopodoviruses and cyanomyoviruses in CR and HR indicates gene sharing between marine and freshwater cyanophages. This finding provides new insights into the evolutionary relationship between freshwater and marine cyanophage communities. Contrary to the dissimilarities that has been observed between freshwater and marine cyanophage isolates, our discovery shows that some freshwater cyanophages are closer evolutionarily to their marine counterparts, as indicated by them sharing the majority of core genes, especially for cyanophages infecting picocyanobacteria. The genetic distinctness between freshwater and marine cyanophage phages could be largely due to the lack of cyanophages infecting a common host species. To the best of our knowledge, out of the 22 freshwater cyanophage isolates sequenced previously, only 3 isolates infect picocyanobacteria (S-CRM01, S-EIV1, and S-LBS1). Yet, the majority of marine cyanophage isolates infect *Synechococcus* spp. and *Prochlorococcus* spp. With more marine cyanophages infecting cyanobacteria than *Synechococcus* spp. and *Prochlorococcus* spp., a clearer evolutionary relationship between freshwater and marine cyanophages needs to be elucidated.

In conclusion, this study describes the characteristics and genomes of S-SRP01 and S-SRM01, two freshwater cyanophages that are highly similar to their marine counterparts despite being isolated from a freshwater lake. Our discovery reveals significant gene sharing between freshwater and marine cyanophage isolates. This result is further expanded by metagenomic identification of T4-like and T7-like cyanophages sharing core genes with marine counterparts in freshwater viral metagenomes. Our results indicate implications in the evolutionary relationships between freshwater and marine environments.

## MATERIALS AND METHODS

### Host cells.

The nonaxenic *Synechococcus* sp. strain SR-R4S1 (infected by S-SRP01) and *Synechococcus* sp. strain SR-C6 (infected by S-SRM01) were isolated in February 2019 from a tropical eutrophic freshwater body that is adjacent to the sea but separated by a dam (see Fig. S1 in the supplemental material). The lake was previously an estuary and converted into a freshwater lake by damming the mouth of the estuary with a tidal gate about 10 years ago. Lake water is discharged occasionally into the sea when the upstream water level is high due to storm events. The mean salinity of the lake is approximately 0.139‰ (± 0.028), which is representative of the salinity in a freshwater body (i.e., 0.5‰ or less) (48). On the other hand, estuaries typically have salinity higher than 0.500‰. The strains were isolated by micropipetting an aliquot from a surface water sample into sterile MLA medium (49) at 25°C. Upon isolation, the isolates were then incubated and maintained in batch culture at 25°C under low irradiance (20 μmol photons m^−2^s^−1^) with a 12-h/12-h light/dark cycle. Identification of the strains was determined through whole-genome sequencing (47). The phylogenetic tree based on 16S rRNA sequence revealed that SR-C6 and SR-R4S1 are present in a mixed-cluster where freshwater and marine *Synechococcus* strains coexist (47).

### Cyanophages.

Cyanophage S-SRP01 and S-SRM01 were isolated from viral concentrates collected from surface water as described above. Briefly, viral concentrate was prepared by filtering 450 ml of water through 0.2-μm-pore size filters (Nucleopore; Merck Millipore) followed by ultrafiltration with 100-kDa molecular weight (MW) cutoff ultrafiltration centrifugal tubes (Amicon Ultra-15 Centrifugal Filter Units; Merck Millipore). Ten-microliter aliquots of viral concentrates were added to exponentially growing cultures of host cyanobacteria in 96-well plate and incubated at 28°C under low radiance (20 μmol photons m^−2^s^−1^) with a 12-h/12-h light/dark cycle for 14 days. A similar irradiance level and light/dark cycles are used commonly in the cultivation and isolation of freshwater cyanophages (20, 50, 51). Culture lysis was determined by more than a 50% decrease in host population measured by flow cytometry using a CytoFLEX flow cytometer (Beckman Coulter Inc., CA, USA). The discriminator was set on forward scatter height (FSC-H) and allophycocyanin height (APC-H), and samples (1 ml) were analyzed at a rate of 30 μl/min (see Fig. S2 in the supplemental material). Next, 1.0-μm FluoSpheres microsphere beads (Thermo Fisher Scientific Inc., USA) were added for absolute counting. A clonal viral isolate was obtained by three rounds of extinction dilution (52) in 96-well microtiter plates.

### Cyanophage amplification and purification.

Cyanophage amplification and purification were carried out as described previously (13). Briefly, 1% (vol/vol) of the virus isolate was added to 30-ml cultures of host cyanobacteria to produce enough phage progeny for the subsequent analysis. A total of 30 ml of the lysate was concentrated and used for DNA extraction to obtain a minimum of 200 ng of phage DNA which is required for phage whole-genome sequencing. DNA concentration was measured by the Qubit DNA high-sensitivity (HS) assay (Thermo Fisher Scientific). When lysis occurred, the lysates were centrifuged at 15,000 × *g* for 5 min to remove cellular debris. The supernatant containing the majority of viral particles was filtered through a 0.22-μm syringe filter (Minisart Syringe Filter, Satorius) to remove cellular debris.

### One-step growth curve and adsorption assay.

To examine the growth of the host under infection, purified phage was added to 30 ml of exponentially growing cultures of host cyanobacteria in a culture flask at a multiplicity of infection (MOI) of ∼8 (S-SRP01) and ∼4 (S-SRM01). A total of 1 ml of the MLA medium was added to another culture to serve as the control. Biological triplicates of phage-added and control treatments were performed. Host cyanobacteria abundance was measured by flow cytometry as described above. To enumerate free phage abundance, 1-ml samples were taken at 0, 2, 4, 6, 8, 10, 12, and 24 hours after phage inoculation and filtered through a 0.2-μm polycarbonate (PC) membrane (Isopore; Millipore). The membrane was washed 3 times with MLA medium to ensure all free phages were collected in the filtrate. A total of 200 μl of the filtrate was used for viral DNA extraction with QIAamp DNA minikit. Five microliters of RNase A was added in the first step to remove free RNA. Free phage DNA was quantified using qPCR with new primers listed in Table S1 in the supplemental material. These primers were designed by uploading major capsid protein gene sequences of each isolate onto Primer-BLAST as the PCR template and default primer parameters were chosen. Primer specificity checking was also performed against the nonredundant (nr) database to ensure that the primers were specific to the template and no other sequences in the nr database could be amplified by the primer sets (53). For the qPCR, the 20-μl qPCR mix contained 10 μl of FastStart universal probe master (Rox), 1 μM each primer, 3 μl of nuclease free water, and 5 μl of DNA template. Thermal cycling was conducted in a StepOnePlus real-time PCR system (Applied Biosystem) with the following program: 10 min denaturation at 95°C, followed by 40 cycles of denaturation at 95°C for 30s, annealing at 58°C for 30s, and extension at 72°C for 30s. Six 10-fold serial dilution standards (ranging from 4.7 × 10^6^ to 4.7 × 10^1^ molecules) were run in triplicates with 2 negative-control reaction mixtures containing 5 μl of nuclease-free water. The standards were prepared by extracting viral DNA from a cyanophage lysate of known titer.

### Transmission electron microscopy.

A total of 30 ml of purified lysate was centrifuged at 5,000 × *g* with 100-kDa MW cutoff ultrafiltration centrifugal tubes (Amicon Ultra-15 centrifugal filter units; Millipore) to increase the phage particle concentration. For staining, 20 μl of gadolinium triacetate (1% [wt/wt]) was adsorbed to the surface of copper grids at room temperature for 1 min. Excess liquid was blotted off from the side of the copper grid with clean filter paper. The grids were viewed and photographed on a JEOL JEM-2100F field emission gun transmission electron microscope at National University of Singapore Faculty of Material Science and Engineering.

### Host range.

Cyanophage infectivity was tested against local freshwater isolates of cyanobacterium strains as well as cyanobacteria obtained from an overseas culture collection. A total of 0.02 ml of the phage lysate was added to cultures of exponentially growing cyanobacteria listed in Table 1 in a 96-well plate. For each cyanobacterium strain tested, 1 well was inoculated with 0.02 ml of MLA medium to serve as a control, while 6 wells were inoculated with the phage lysate. Infectivity was determined by a 50% decline in optical density (OD) reading compared with the control (54).

### DNA extraction, purification, and sequencing.

Host cyanobacteria were grown in 70 ml of MLA medium at 25°C under low irradiance (20 μmol photons m^−2^s^−1^) with 12-h/12-h light/dark cycle until lysis. The lysates were purified as described above. In order to remove free nucleic acids, the lysate was treated with DNase I. The treated lysate was concentrated with 100-kDa MW cutoff ultrafiltration centrifugal tubes (Amicon Ultra-15 centrifugal filter units; Millipore) at 5,000 × *g* to a final volume of 0.2 ml. The QIAamp DNA minikit was used to extract viral DNA with 5 μl of RNase A added in the first step to remove RNA. The cyanophage genome was sequenced using an Illumina high-throughput sequencer, with a 150-bp paired-end library constructed using a New England BioLabs (NEB) Next Ultra DNA library prep kit.

### Genome assembly and annotation.

The sequencing data were trimmed using BBDuk (V38.18) to remove adaptors and Phix reads. Reads were *de novo* assembled into contigs by the Megahit genome assembler (V1.2.9) in meta-sensitive mode (55). The whole-genome sequence of the phage has been submitted to GenBank under accession MW015080 and MW015081. The open reading frames (ORFs) were predicted using Prodigal (V2.6.3) in meta mode (56). Homology searching was performed with Diamond (V0.9.14.115) (57) against the NCBI nonredundant (nr) database (accessed in July 2020). Sequences with E values of <10^−5^ were considered homologs. HHpred analysis against the protein data bank (PDB) and Pfam database were used to predict more distant homologs (58). tRNAscan-SE 2.0 (59) was used to predict tRNA genes. The genomic map was generated with the CGview applet (60).

### Phylogenetic analysis.

For the phylogenetic analysis of S-SRP01, concatenation of the terminase, DNA polymerase, major capsid protein, and exonuclease genes were compared phylogenetically with those from other cyanopodoviruses (see Table S2 in the supplemental material) using Mega-X software (V10.1.6) (61). For phylogenetic analysis of S-SRM01, concatenation of terminase, DNA polymerase, tail sheath, and exonuclease genes were compared phylogenetically with those from other cyanomyoviruses (Table S2). ClustalX was used to align the inferred amino acid sequences with default parameters (62). Based on the multiple sequence alignment, the Jones-Taylor-Thornton (JTT) model was selected and the maximum likelihood tree was constructed with 100 bootstrap replicates (63). S-SRP01 and S-SRM01 genome sequences were also uploaded to the ViPTree server to construct the whole-genome viral proteomic tree with 2,689 viral genomes available in the Virus Host database (DB) (64).

### Comparative genomics.

To compare genetic similarities between S-SRP01 and S-SRM01 with other cyanophages, complete genome sequences of cultured cyanopodovirus and cyanomyoviruses were obtained from GenBank (Table S2). To ensure consistent gene calling, Prokka (V1.14.5) was used to reannotate selected cyanophage genomes (65). The resultant GenBank feature file was used as the input file to generate whole-genome protein sequence alignment using Easyfig (V2.2.2) (66).

### Metagenomic identification of marine cyanophages in the freshwater virome.

To find out whether there are freshwater cyanophages displaying significant gene sharing in other parts of the world, viral metagenomic raw reads from previous studies were acquired from NCBI Sequence Read Archive (SRA) (see Table S3 in the supplemental material) (67–71). SRA files were first converted to fastq format before being trimmed with BBDuk (V38.18) to remove adaptors and Phix reads. *De novo* assembly of the trimmed data set was performed with MetaSPAdes V3.13.0 (72) with only contigs longer than 10,000 bp kept for further analysis. Open reading frames of the assembled contigs were predicted with Prodigal (56). Assembled contigs were annotated preliminary with Diamond (57) against protein sequences of all cultured marine cyanopodovirus as database. This procedure serves as an ultrafast way to find out possible cyanopodoviruses in the target metagenomic data. Contigs with significant homology to marine cyanopodovirus genes (no less than 10 homologs with E values of <10^−5^) were selected for annotation against the NCBI nr database and whole-genome protein sequence alignment with other cyanopodoviruses. A similar analysis was carried out for cyanomyoviruses. Unfortunately, due to the dissimilarity among cyanosiphoviruses and a lack of core genes (30), we were unable to identify marine cyanosiphoviruses present in freshwater environments.

### Data availability.

All viral metagenomic data used in this study are available openly in the Sequence Read Archive (SRA) under the accession numbers listed in Table S3. Genomes of S-SRP01 and S-SRM01 are available in the NCBI database through accession numbers MW015080 and MW015081.
