# Supplementary material for: Novel Freshwater Cyanophages Provide New Insights into Evolutionary Relationships between Freshwater and Marine Cyanophages
Source: Microbiol Spectr. 2021 Sep 29;9(2):e00593-21. doi: 10.1128/Spectrum.00593-21 (PMC8557907; doi:10.1128/Spectrum.00593-21)
Supplement: SUPPLEMENTAL FILE 1 — Supplemental material. Download SPECTRUM00593-21_Supp_1_seq11.pdf, PDF file, 0.7 MB [file spectrum00593-21_supp_1_seq11.pdf]

Supplementary table S1 list of primers used for quantifying S-SRP01 and S-SRM01

Target gene	Primer	Sequence 5’ to 3’
S-SRP01 major capsid protein	R4S1F	CAC TAC AGC CTG CGT TCT GA
	R4S1R	CAC CCA GTT GGA TCA CCG AA
S-SRM01 major capsid protein	C6F	TGT TCC TGG TGC TCA GGT CA
	C6R	TTA GCG TTG AGT GCA GGG G

Supplementary table S2 list of cyanophages used for phylogenetic and comparative genomic analysis

Accesion	Organism	Type
NC_024358.1	Anabaena phage A-4L	Podoviridae
NC_020865.1	Cyanophage KBS-P-1A	Podoviridae
NC_022751.1	Cyanophage PP	Podoviridae
NC_016656.1	Cyanophage P-SSP2	Podoviridae
NC_020872.1	Cyanophage SS120-1	Podoviridae
NC_009531.1	Cyanophage Syn5	Podoviridae
NC_009551.1	Phormidium phage Pf-WMP3	Podoviridae
NC_008367.1	Phormidium phage Pf-WMP4	Podoviridae
NC_020878.1	Prochlorococcus phage P-GSP1	Podoviridae
HQ332139.1	Prochlorococcus phage P-RSP2	Podoviridae
NC_020835.1	Prochlorococcus phage P-SSP10	Podoviridae
NC_020874.1	Prochlorococcus phage P-SSP3	Podoviridae
NC_006882.2	Prochlorococcus phage P-SSP7	Podoviridae
NC_003390.2	Synechococcus phage P60	Podoviridae
NC_025456.1	Synechococcus phage S-CBP1	Podoviridae
NC_025455.1	Synechococcus phage S-CBP2	Podoviridae
NC_025461.1	Synechococcus phage S-CBP3	Podoviridae
NC_025464.1	Synechococcus phage S-CBP4	Podoviridae
KJ410740.1	Synechococcus phage S-EIVl	Podoviridae
NC_020867.1	Synechococcus phage S-RIP1	Podoviridae
NC_020838.1	Synechococcus phage S-RIP2	Podoviridae
KU234533	Nostoc phage A1	Myoviridae
NC_008562	Microcystis phage Ma-LMM01 DNA	Myoviridae
NC_029002	Microcystis phage MaMV-DC	Myoviridae

NC_020845	Cyanophage MED4-213	Myoviridae
KU234532	Nostoc phage N1	Myoviridae
NC_015280	Prochlorococcus phage P-HM1	Myoviridae
NC_015284	Prochlorococcus phage P-HM2	Myoviridae
NC_021071	Cyanophage P-RSM1	Myoviridae
HQ634176	Cyanophage P-RSM3	Myoviridae
NC_015283	Prochlorococcus phage P-RSM4	Myoviridae
NC_020855	Cyanophage P-RSM6	Myoviridae
NC_006883	Prochlorococcus phage P-SSM2	Myoviridae
NC_021559	Prochlorococcus phage P-SSM3	Myoviridae
NC_006884	Prochlorococcus phage P-SSM4	Myoviridae
HQ632825	Prochlorococcus phage P-SSM5	Myoviridae
NC_015290	Prochlorococcus phage P-SSM7	Myoviridae
NC_015569	Synechococcus phage S-CRM01	Myoviridae
NC_006820	Synechococcus phage S-PM2	Myoviridae
NC_013085	Synechococcus phage S-RSM4	Myoviridae
NC_015281	Synechococcus phage S-ShM2	Myoviridae
NC_015282	Synechococcus phage S-SM1	Myoviridae
NC_015279	Synechococcus phage S-SM2	Myoviridae
JF974292	Cyanophage S-SSM2	Myoviridae
NC_020875	Cyanophage S-SSM4	Myoviridae
NC_015289	Synechococcus phage S-SSM5	Myoviridae
NC_015287	Synechococcus phage S-SSM7	Myoviridae
NC_015288	Prochlorococcus phage Syn1	Myoviridae
HQ634191	Cyanophage Syn10	Myoviridae
NC_015286	Synechococcus phage Syn19	Myoviridae
HQ634190	Cyanophage Syn2	Myoviridae
NC_021072	Cyanophage Syn30	Myoviridae
NC_015285	Prochlorococcus phage Syn33	Myoviridae
NC_008296	Synechococcus phage syn9	Myoviridae

Supplementary table S3 List of SRA accession used for metagenomic analysis

SRA Accesion	Description	Reference
SRR8075992	Chattahoochee river	(1)
SRR8075980	Chattahoochee river	
SRR8075978	Chattahoochee river	
SRR8075991	Chattahoochee river	
SRR5989993	Cochin Estuary	(2)

SRR5989996	Cochin Estuary	
SRR5989994	Cochin Estuary	
SRR5989995	Cochin Estuary	
ERR019477	French lake	(3)
ERR019478	French lake	
ERR1823950	Hanriver GunamB	(4)
ERR1823951	Hanriver Daesung	
ERR1823952	Hanriver PaldangB	
ERR1823953	Hanriver HannamB	
ERR1823954	Hanriver IlsanB	
ERR1824253	Hanriver HaejungB	
SRR4449268	Lough neagh	(5)
SRR5936590	Lake Baikal	

Supplementary table S4 Predicted ORFs of cyanophage S-SRP01 with similarity to genes of known function

ORF	Genebank id	% identity	E-value	Putative protein encoded	Organism
3	YP_009103500.1	88	9.40E-308	DNA maturase B (terminase)	Synechococcus phage S-CBP3
4	YP_009103816.1	74.1	1.80E-83	thymidylate synthase	Synechococcus phage S-CBP4
15	YP_009103173.1	41	1.40E-55	integrase	Synechococcus phage S-CBP1
16	YP_007673180.1	59.2	1.20E-265	RNA polymerase	Synechococcus phage S-RIP2
17	YP_009822226.1	71	4.20E-68	ssDNA binding protein	Synechococcus phage S-CBP4
18	YP_009103783.1	83.8	2.60E-52	endonuclease	Synechococcus phage S-CBP4
21	NBS71251.1	81.6	1.20E-200	toprim domain-containing protein, partial	bacterium
23	OUU96656.1	66.5	8.30E-63	T7-like DNA polymerase 3'-5' exonuclease	uncultured phage_MedDCM-OCT-S45-C4

24	ABU50234.1	64.9	2.00E-163	DNA polymerase	Cyanophage S-CBP3
27	YP_009103521.1	61.3	1.40E-85	exonuclease	Synechococcus phage S-CBP3
29	YP_009103134.1	59.5	5.10E-27	MazG nucleotide pyrophosphohydrolase	Synechococcus phage S-CBP2
30	QDP67893.1	74.3	1.10E-65	putative uncharacterized nin region protein	Prokaryotic dsDNA virus sp.
31	NBS69168.1	88.9	9.80E-244	ribonucleotide reductase	bacterium
32	YP_009103525.1	81.1	9.60E-110	class I ribonucleotide reductase beta subunit	Synechococcus phage S-CBP3
36	YP_009103528.1	93.7	1.10E-273	head-to-tail connector (portal)	Synechococcus phage S-CBP3
37	YP_009103529.1	75.1	1.10E-95	capsid assembly protein (scaffold)	Synechococcus phage S-CBP3
38	WP_021692686.1	64.9	3.70E-26	septal ring lytic transglycosylase RlpA family protein	Limimaricola cinnabarinus
43	NBS70089.1	91.2	3.50E-166	T7 capsid protein	bacterium
45	YP_009103534.1	80.8	3.00E-97	tail tubular protein A	Synechococcus phage S-CBP3
46	YP_009103535.1	83.7	0.00E+00	tail tubular protein B	Synechococcus phage S-CBP3
48	YP_009103195.1	23.9	2.4e-51	Internal core protein	Synechococcus phage S-CBP1
56	YP_009778077.1	72.2	2.30E-42	possible endonuclease	uncultured phage_MedDCM-OCT-S28-C3

Supplementary table S5 Predicted ORFs of cyanophage S-SRM01 with similarity to genes of known function

ORF	Genebank id	% identity	E-value	Putative protein encoded	Organism
1	BAR36264.1	40.2	8.40E-89	baseplate wedge initiator	uncultured Mediterranean phage uvMED
4	BAQ89940.1	32.9	8.70E-23	putative endosialidase	uncultured Mediterranean phage uvMED
6	BAR32982.1	47.4	1.70E-07	phage tail fiber-like protein	uncultured Mediterranean phage uvMED
7	BAR34239.1	44.4	2.80E-20	virion structural protein	uncultured Mediterranean phage uvMED
8	BAR31382.1	34.1	1.20E-12	putative endosialidase	uncultured Mediterranean phage uvMED
11	QCW22993.1	32.5	2.10E-17	endosialidase	Synechococcus phage S-B05
13	NBU96809.1	83.8	2.70E-32	glutaredoxin	Spirochaetia bacterium
22	YP_009323193.1	71.3	8.90E-98	thymidylate synthase	Synechococcus phage S-CAM7
25	YP_009810812.1	76.8	5.80E-113	phosphate starvation-inducible protein	Synechococcus phage S-T4
26	YP_214232.1	76.5	2.70E-98	exonuclease	Prochlorococcus phage P-SSM2
27	YP_214233.1	77.9	1.10E-34	late promoter transcription accessory protein	Prochlorococcus phage P-SSM2
28	YP_214238.1	69.9	7.50E-76	loader of gp41 DNA helicase	Prochlorococcus phage P-SSM2
30	CAB4154594.1	44.4	9.20E-35	Nuclease associated modular domain 3	uncultured Caudovirales phage

31	NBW56880.1	80.9	6.10E-141	single-stranded DNA-binding protein	bacterium
32	YP_214241.1	46	6.10E-53	base plate wedge component	Prochlorococcus phage P-SSM2
33	AOV62195.1	38.3	8.80E-44	baseplate tail tube cap	Synechococcus phage S-CAM7
36	YP_214244.1	66.9	7.30E-52	head completion protein	Prochlorococcus phage P-SSM2
38	NBW56886.1	87.9	2.10E-112	baseplate protein	bacterium
41	RPJ57726.1	27.3	1.00E-33	M23 family metallopeptidase	Dehalococcoidia bacterium
43	YP_214251.1	32.7	6.70E-76	T4-like baseplate hub and tail lysozyme	Prochlorococcus phage P-SSM2
50	YP_214265.1	47	0.00E+00	putative tail fiber	Prochlorococcus phage P-SSM2
52	CAB5222023.1	38.6	1.90E-88	Flavin-dependent halogenase	uncultured Caudovirales phage
55	NBP56166.1	70.7	5.80E-171	DEAD/DEAH box helicase	bacterium
75	CAB4125199.1	58.3	3.30E-22	Nuclease associated modular domain 3	uncultured Caudovirales phage
114	CAB4125199.1	60.6	1.50E-23	translation initiation factor 2	Synechococcus phage B3
116	NJL22330.1	50	4.30E-28	peptide deformylase	Leptolyngbyaceae cyanobacterium SM1_3_5
122	AIX28551.1	43.7	4.70E-33	2OG-Fe(II) oxygenase	Synechococcus phage ACG-2014j
123	HAF00099.1	25.7	1.70E-11	TPA: hypothetical protein DCG63_02270	Methylophilaceae bacterium

130	NBP03544.1	47.5	2.20E-50	AbrB/MazE/SpoVT family DNA-binding domain-containing protein	Proteobacteria bacterium
135	YP_007006052.1	62.9	3.70E-17	CP12 protein	Cyanophage S-TIM5
139	WP_126146876.1	47.1	1.00E-23	membrane protease subunit	Synechococcus elongatus
179	BAR36583.1	67.6	7.30E-89	Phage protein Gp37/Gp68-like	uncultured Mediterranean phage uvMED
180	WP_171382129.1	42.4	2.30E-21	HNH endonuclease	Vibrio europaeus
185	NBW41495.1	57.1	1.90E-11	AbrB/MazE/SpoVT family DNA-binding domain-containing protein	bacterium
190	NBU81178.1	83.3	2.40E-47	adenosylmethionine decarboxylase	Flavobacteriaceae bacterium
192	AIX42332.1	67.9	1.10E-41	base plate wedge subunit	Synechococcus phage ACG-2014f
193	YP_007674524.1	63.8	1.20E-226	baseplate wedge subunit	Synechococcus phage S-SKS1
194	YP_214343.1	32.6	0.00E+00	baseplate wedge initiator	Prochlorococcus phage P-SSM2
195	YP_214344.1	57.4	4.00E-169	baseplate wedge	Prochlorococcus phage P-SSM2
196	YP_009810953.1	41.8	0.00E+00	virulence-associated VriC protein	Synechococcus phage S-T4
202	YP_214353.1	53.6	3.30E-114	neck protein	Prochlorococcus phage P-SSM2
203	CAB4133474.1	45.3	1.10E-17	HNHc domain containing protein	uncultured Caudovirales phage
204	BAR32889.1	68.1	3.70E-87	proximal tail sheath stabilization	uncultured Mediterranean

					phage uvMED
205	NBW58016.1	86.4	2.70E-59	terminase small subunit	bacterium
207	CAB4167653.1	46.2	8.70E-23	Recombination endonuclease VII	uncultured Caudovirales phage
210	NBW57912.1	92.9	0.00E+00	phage tail sheath protein	bacterium
211	NBP55557.1	85.4	0.00E+00	terminase large subunit	bacterium
212	YP_214362.1	72.5	1.60E-78	tail tube monomer	Prochlorococcus phage P-SSM2
213	NBU99033.1	90.2	1.00E-247	portal vertex protein of head, partial	Spirochaetia bacterium
216	NDB85248.1	89.8	6.90E-107	primosomal protein	Alphaproteobacteria bacterium
217	NDB82779.1	62.2	1.70E-84	T4 prohead core scaffold protein	Alphaproteobacteria bacterium
218	YP_214367.1	77.4	5.40E-197	precursor of major head subunit	Prochlorococcus phage P-SSM2
220	YP_214369.1	59.6	2.30E-55	head-proximal tip of tail tube tail completion + sheath stabilizer protein	Prochlorococcus phage P-SSM2
221	YP_007674498.1	75.2	2.80E-56	recombination protein UvsY	Synechococcus phage S-SKS1
222	MBN88260.1	84.8	1.40E-248	recombination protein UvsW	Candidatus Woesearchaeota archaeon
223	YP_214375.1	52.6	1.40E-36	methylamine utilization protein	Prochlorococcus phage P-SSM2
224	YP_214376.1	85.9	1.20E-71	sigma factor for late transcription	Prochlorococcus phage P-SSM2

225	NDC49483.1	53.2	7.40E-15	endonuclease VII	Micrococcales bacterium
226	NBP57866.1	85.5	2.80E-179	recombinase	bacterium
228	YP_214379.1	62.7	6.40E-192	recombination endonuclease subunit	Prochlorococcus phage P-SSM2
230	MAG49887.1	65.8	5.10E-263	peptidase	Candidatus Woesearchaeota archaeon
232	NBO99060.1	83.9	1.40E-176	MoxR family ATPase	Proteobacteria bacterium
234	NDD52089.1	88.2	1.50E-104	DNA polymerase	Actinobacteria bacterium
238	NDB84469.1	81.2	1.00E-143	AAA family ATPase	Alphaproteobacteria bacterium
241	QBP06016.1	74.6	1.60E-153	putative cytoplasmic protein	Synechococcus phage S-B68
242	NBU47906.1	89.4	7.90E-58	DNA polymerase	Flavobacteriales bacterium
243	NBU47905.1	87	3.30E-68	translation repressor protein	Flavobacteriales bacterium
245	NBU47904.1	83.3	7.10E-64	heat-shock protein	Flavobacteriales bacterium
249	YP_007674467.1	81.5	0.00E+00	DNA polymerase	Synechococcus phage S-SKS1
252	NBW58759.1	94.3	4.90E-176	recombinase RecA	bacterium
254	ACY76067.1	82.8	1.20E-214	DNA primase/helicase	Prochlorococcus phage P-SSM2
255	NBV57733.1	83.1	1.30E-58	pyrophosphatase	Bacteroidetes bacterium
258	NBW57656.1	86.3	8.80E-210	cytidyltransferase	bacterium
265	NBP56667.1	81.1	1.40E-159	DNA primase	bacterium
266	BAR33421.1	82.3	0.00E+00	putative ribonucleotide reductase A subunit	uncultured Mediterranean phage uvMED

267	NDB84431.1	86.1	3.60E-194	ribonucleotide-diphosphate reductase subunit beta	Alphaproteobacteria bacterium
272	NBP55946.1	94.3	5.70E-73	NAD synthetase	bacterium
275	AFK66345.1	61.1	4.00E-48	lysin	Synechococcus phage S-CBM2
278	AFK66348.1	74.3	2.10E-71	gp214	Synechococcus phage S-CBM2
281	NBP55956.1	90	1.00E-18	histone H1	bacterium
283	NBW34632.1	75.5	4.40E-210	porin	Cytophagia bacterium
286	NBX71672.1	56.7	1.00E-37	DNA starvation/stationary phase protection protein	bacterium
289	CAB5222682.1	29.1	1.30E-13	Intramolecular chaperone auto-processing domain containing protein	uncultured Caudovirales phage
296	WP_053078075.1	33.5	9.30E-32	glycosyltransferase	Parabacteroides goldsteinii
298	AIX18518.1	76.7	3.60E-147	GDP-D-mannose 4,6-dehydratase	Synechococcus phage ACG-2014f
299	NDB85746.1	84.2	4.70E-149	GDP-L-fucose synthase, partial	Alphaproteobacteria bacterium
300	NBW20991.1	52.4	9.30E-47	SIS domain-containing protein	Caulobacteraceae bacterium
301	RPI62791.1	77.4	1.30E-241	NDP-sugar synthase	Ignavibacteriales bacterium
307	YP_004322340.1	55.4	1.20E-76	putative transferase	Synechococcus phage S-SM2
308	HCX21903.1	64.4	3.10E-72	TPA: FkbM family methyltransferase	Cytophagales bacterium
309	YP_009810865.1	62.5	3.40E-98	alpha-1,2-fucosyltransferase	Synechococcus phage S-T4

311	NBW34634.1	59.9	2.10E-89	alpha-1,2-fucosyltransferase	Cytophagia bacterium
312	NDB84720.1	79.8	9.50E-127	glycosyltransferase family 25 protein	Alphaproteobacteria bacterium
314	NDB87304.1	79	5.00E-21	high light inducible protein	Alphaproteobacteria bacterium
315	ARW57067.1	92.7	4.90E-44	ferredoxin	Synechococcus phage S-H35
316	NDB86985.1	74.4	3.90E-08	high light inducible protein	Alphaproteobacteria bacterium
317	NBW34626.1	84.7	2.00E-83	peroxiredoxin	Cytophagia bacterium
319	NBP04124.1	89.1	2.70E-39	photosystem II D1 protein, partial	Proteobacteria bacterium
320	YP_636209.1	40.7	3.30E-30	putative site-specific DNA endonuclease (chloroplast)	Tupiella akineta
321	AGL79224.1	97.5	3.00E-86	photosystem II D1 protein, partial	uncultured organism
322	YP_195212.1	83.1	4.30E-65	homing endonuclease	Synechococcus phage S-PM2
324	NDD82610.1	87.6	1.50E-123	UDP-2,3-diacylglucosamine diphosphatase	Verrucomicrobia bacterium
328	NBP02823.1	78	2.90E-57	HNH endonuclease	Proteobacteria bacterium
334	AXN58399.1	57.4	4.80E-18	putative tail fiber protein	Synechococcus virus S-PRM1
338	NBV28374.1	60.7	9.50E-159	tetratricopeptide repeat protein	bacterium
345	NBV28439.1	81.9	1.20E-124	sulfotransferase	bacterium
347	NBV28437.1	68.6	2.50E-123	glycosyltransferase	bacterium
349	YP_004322338.1	87.3	2.40E-178	nucleotide-sugar epimerase	Synechococcus phage S-SM2

Supplementary table S6 viral contigs assembled from freshwater metagenome

Contigs	coverage	Assembled from
HR_podo contig 1	13.6	HanRiver
HR_podo contig 2	9.2	HanRiver
CR_podo contig 1	106.5	Chattahochee River
CR_podo contig 1	82.2	Chattahochee River
HR_myo contig 1	23.5	HanRiver
HR_myo contig 2	43.7	HanRiver

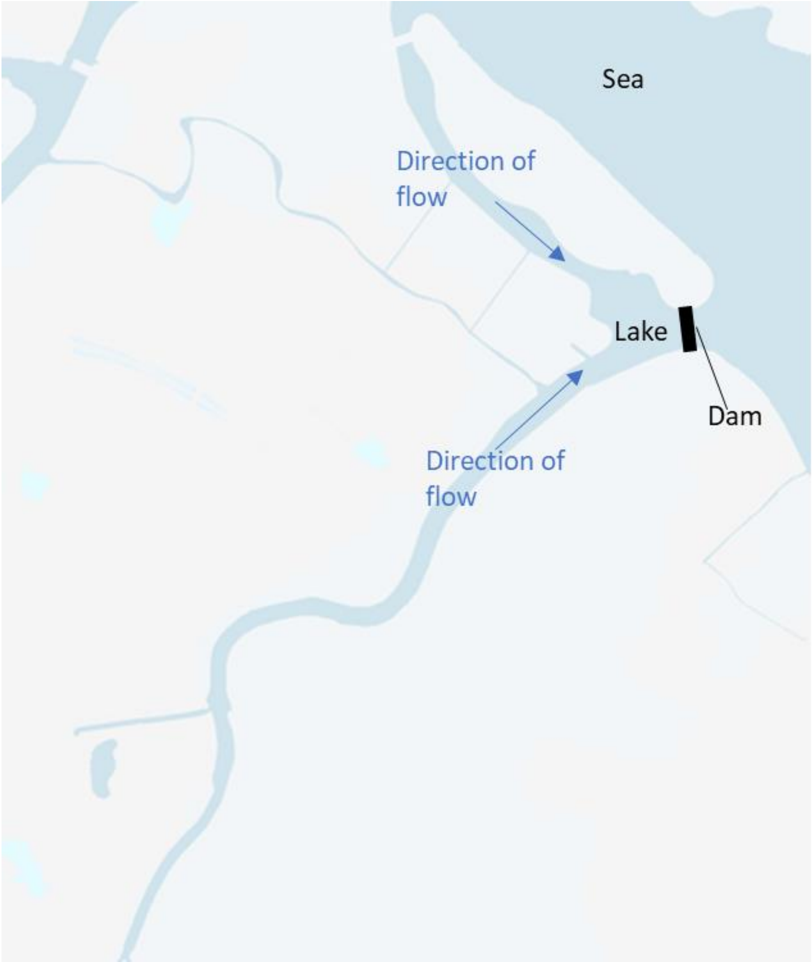

Supplementary Figure S1 Map of the isolation site. The sampling site is located in Singapore at Latitude:1.40° Longitude: 103.927°

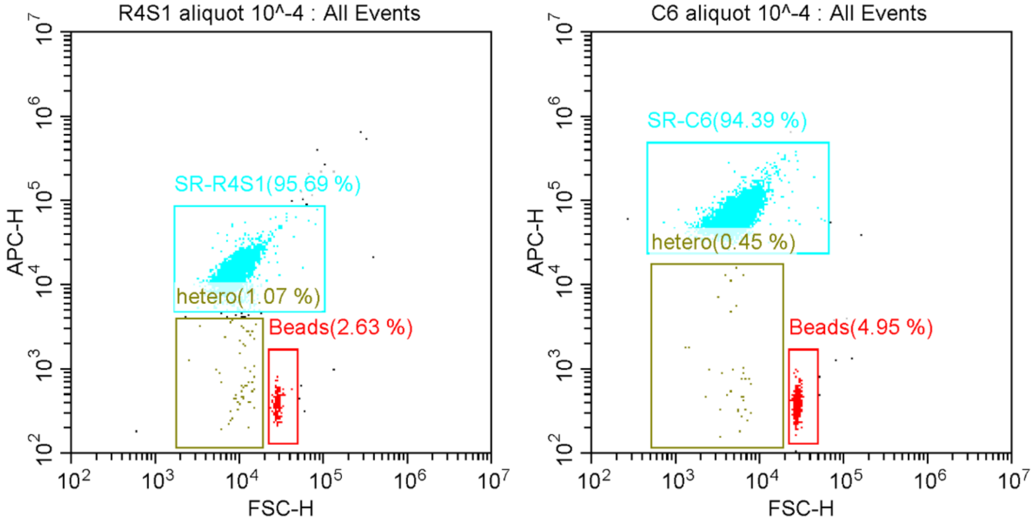

Supplementary Figure S2 Enumeration of cyanobacteria host using flow cytometry. The culture is dominated by cyanobacteria and the heterotrophic population can be readily separated from the cyanobacteria population based on differing levels of Allophycocyanin signal. Therefore, it's unlikely that the heterotrophic bacteria have observable impacts on the host growth curves.

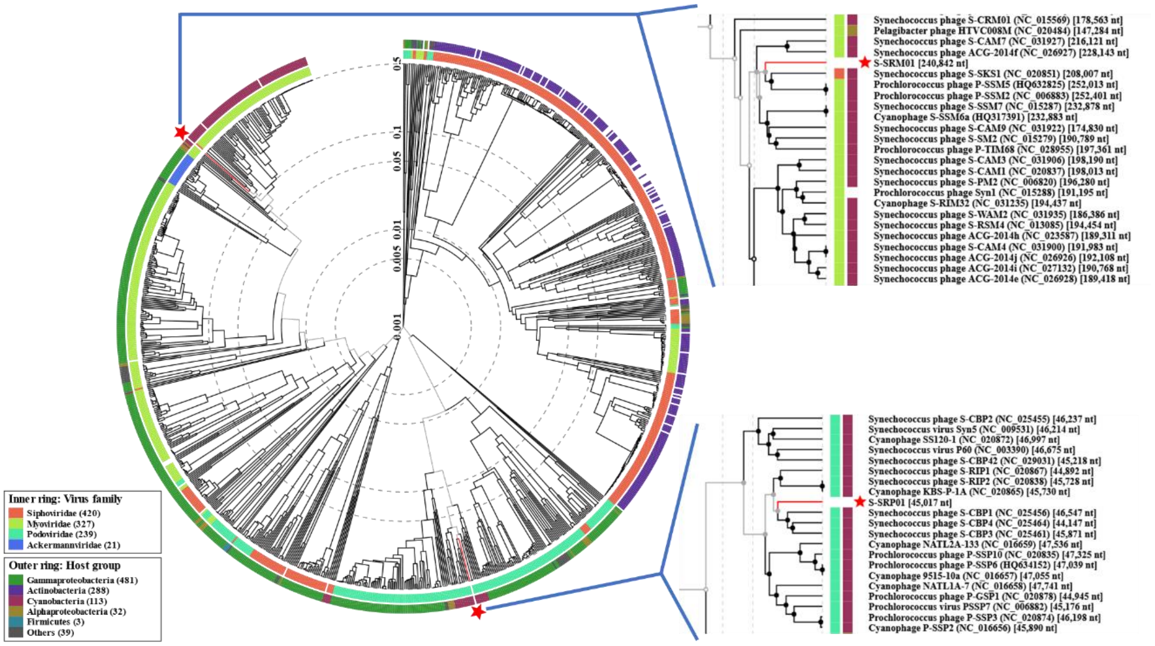

Supplementary Figure S3 Viptree whole genome viral proteomic tree of S-SRP01 and S-SRM01 with related viruses

1. Ruiz-Perez CA, Tsementzi D, Hatt JK, Sullivan MB, Konstantinidis KT. 2019. Prevalence of viral photosynthesis genes along a freshwater to saltwater transect in Southeast USA. Environ Microbiol Rep 11:672-689.
2. Jasna V, Parvathi A, Dash A. 2018. Genetic and functional diversity of double-stranded DNA viruses in a tropical monsoonal estuary, India. Scientific Reports 8:16036.
3. Roux S, Enault F, Robin A, Ravet V, Personnic S, Theil S, Colombet J, Sime-Ngando T, Debroas D. 2012. Assessing the diversity and specificity of two freshwater viral communities through metagenomics. PloS one 7:e33641-e33641.
4. Moon K, Jeon JH, Kang I, Park KS, Lee K, Cha C-J, Lee SH, Cho J-C. 2020. Freshwater viral metagenome reveals novel and functional phage-borne antibiotic resistance genes. Microbiome 8:75-75.
5. Arkhipova K, Skvortsov T, Quinn JP, McGrath JW, Allen CCR, Dutilh BE, McElarney Y, Kulakov LA. 2018. Temporal dynamics of uncultured viruses: a new dimension in viral diversity. The ISME Journal 12:199-211.
